# Clinical Insights Into Waardenburg-Shah Syndrome: A Case Series and Literature Review

**DOI:** 10.7759/cureus.59858

**Published:** 2024-05-08

**Authors:** Sri Meghana Kankipati, Akhshaya Mahalingam, Aisha Reshie, Falah Fayaz, Simran Nimal, Dileep Duggineni

**Affiliations:** 1 Medicine and Surgery, Andhra Medical College, Visakhapatnam, IND; 2 Medicine and Surgery, Stanley Medical College, Chennai, IND; 3 Internal Medicine, Government Medical College, Srinagar, IND; 4 Medicine and Surgery, Byramjee Jeejeebhoy (BJ) Government Medical College, Pune, IND; 5 Internal Medicine, Siddhartha Medical College, Vijayawada, IND

**Keywords:** waardenburg-shah syndrome, subtype 4, inherited disorder, congenital disorder, case series

## Abstract

Researching Waardenburg syndrome (WS) underscores its rarity and complex symptomatology, presenting as a congenital disorder predominantly inherited in an autosomal dominant pattern. It exhibits incomplete penetrance, which results in a wide range of clinical manifestations, with variable phenotypic presentations within the same family as well. The most commonly found features are facial abnormalities, hypopigmentation of the skin, heterochromia iridis, and conductive deafness. Adding to the eccentricities of this syndrome are its four subtypes, each presenting with its specific clinical features, which helps in delineating the subtype. A mutated paired box 3 (*PAX3*​​​​​) gene manifests as type 1 Waardenburg, which is characterized by sideways displacement of the inner angles of the eyes (i.e., dystopia canthorum), widely spaced eyes, congenital sensorineural hearing impairment, and patchy pigmentation of the iris, skin, and hair. Due to insufficient research, it has been difficult to isolate all the genetic mutations responsible for type 2, but its phenotype is very similar to type 1 with minor differences. Type 3 is characterized by musculoskeletal abnormalities. Waardenburg-Shah syndrome (type 4), which is associated with Hirschsprung disease, is the rarest subtype and is caused by genetic mutations in the endothelin receptor type B (*EDNRB*), endothelin-3 (*EDN3*), or sex-determining region Y (SRY) box 10 (*SOX10*) gene. We present a case series of this unique subtype that presented with a typical history of constipation due to Hirschsprung disease and had phenotypic manifestations of white forelock, heterochromia iridis, and bilateral sensorineural hearing loss (SNHL). In parallel with a positive 1° family history of a white forelock, we reflect on the fundamentals of this unique syndrome, as well as its management protocols, highlighting the importance of genetic counseling and cultivation of a high index of suspicion for its diagnosis.

## Introduction

Waardenburg syndrome (WS) is an auditory-pigmentary syndrome caused by abnormal neural crest migration, broadly classified into four subtypes. Type 1 WS is characterized by the presence of dystopia canthorum, complete or partial heterochromia iridis, sensorineural hearing loss (SNHL), and abnormal pigmentation of hair and skin [1]. Waardenburg consortium criteria have five major and six minor criteria, and the diagnosis of type 1 WS is made if two major or one major and two minor criteria are met [2]. Major criteria include congenital sensorineural hearing loss, abnormal pigmentation of the iris, white forelock, dystopia canthorum (assessed by W index derived by discriminant analysis using inner canthal, interpupillary, and outer canthal distances), and affected first-degree relative [3]. Minor criteria include congenital leucoderma, synophrys, broad and high nasal bridge, hypoplastic alae nasi, and premature greying of hair. The importance of dystopia canthorum is striking (as assessed by the W index). Values more than 1.95 have been associated with paired box 3 (*PAX3​​​​​*) gene abnormalities, while those below 1.95 do not show *PAX3* involvement [4]. Type 2 WS comprises all the features of type 1 WS except for dystopia canthorum; the diagnostic criteria for type 2 WS consist of any two major criteria (without considering dystopia canthorum) and include premature greying as one of the major criteria. Type 3 WS, also known as Klein-Waardenburg syndrome, is akin to type 1 with additional musculoskeletal deformities. Klein described findings similar to that of type 1 WS, associated with an amyoplasia-like condition in his patient [5].

Shah et al. described 12 cases among five families in Bombay with WS associated with Hirschsprung disease identified during autopsy, which was later identified as type 4 WS, also known as Waardenburg-Shah syndrome. This type is characterized by oculocutaneous findings similar to that of type 2 WS, along with Hirschsprung disease manifesting as parasympathetic aganglionosis of the colon [6]. Waardenburg-Shah syndrome is an autosomal recessive disorder with mutations identified in the sex-determining region Y (SRY) box 10 (*SOX10*), endothelin-3 (*EDN3*), or its receptor endothelin receptor type B (*EDNRB*) genes, which play a vital role in neural crest cell migration. Other types of WS are also associated with *PAX3* and microphthalmia-associated transcription factor (*MITF*) gene mutations [7].

The incidence of Waardenburg syndrome is around 1/212,000, with autosomal dominant or recessive inheritance with 20% penetrance. Out of this, 19% are type 4 WS [8]. There are less than 100 cases of type 4 WS reported in the literature, making Waardenburg-Shah syndrome very rare [9]. The diagnosis of Waardenburg-Shah syndrome is challenging owing to its rarity and variable phenotypic expression. Clinical history and physical examination along with imaging for Hirschsprung disease in patients with congenital deafness (as diagnosed with brainstem auditory evoked response (BAER)) and piebaldism can help diagnose patients with type 4 WS [10]. Rectal biopsy and genetic testing can aid in definitive diagnosis [11]. Rectal biopsy demonstrating aganglionosis is important, especially in cases with extensive involvement in whom imaging may not be of much help. The clinical course is influenced by the extent of Hirschsprung disease, which in turn changes management and surgical technique selection. Patients undergoing surgical correction using different pull-through techniques (Soave, Swenson, and Duhamel procedures) for Hirschsprung disease have postoperative events similar to that of short bowel syndromes consisting of dyselectrolytemia, bacterial overgrowth, total parenteral nutrition (TPN), and catheter-related complications. Mortality is mainly due to sepsis and hepatic dysfunction [12].

In this case report, we present two cases of type 4 WS in a family and aim to review the pathophysiology of Waardenburg-Shah syndrome and shed light on diagnostic and therapeutic challenges faced when dealing with such a rare oculo-pigmentary disorder in a resource-limited setting.

## Case presentation

Case 1

We report a case of a 38-year-old male who presented with concerns regarding the health of his newborn daughter, who exhibits an unusual eye color and potential hearing difficulties. The patient, who had not previously sought medical attention for any specific health issues, noticed strikingly blue irises in his newborn daughter shortly after her birth. Concerned by this observation, he sought medical advice. The patient and his wife noticed that their daughter seemed to exhibit signs of hearing impairment, prompting further evaluation. The patient reports no significant past medical history. He denies any history of chronic illnesses, surgeries, or hospitalizations. There is no known family history of Waardenburg syndrome or other genetic disorders in the patient's family. Both parents are phenotypically normal. The patient is married and denies tobacco, alcohol, or recreational drug use. He resides in a suburban area with his wife and newborn daughter.

**Figure 1:**
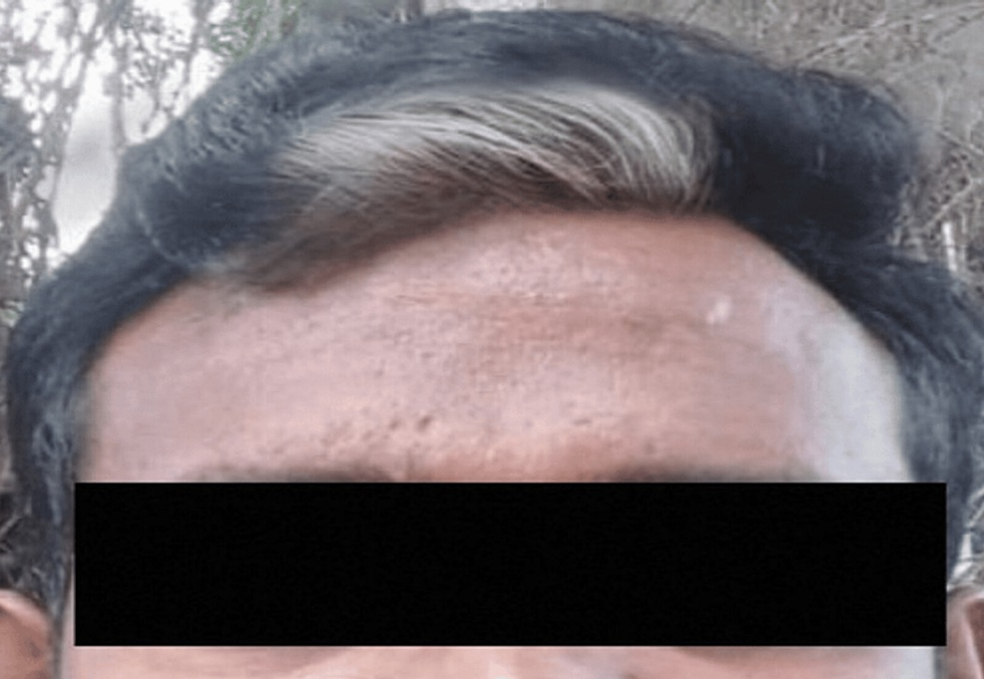
White forelock

The patient denies any recent fevers, chills, weight changes, or any other systemic symptoms. He reports no issues with vision, motor function, or cognitive function. Upon physical examination, the patient appears phenotypically normal except for the white forelock as observed in Figure 1. There are no apparent craniofacial abnormalities, auditory abnormalities, or pigmentary disturbances observed. The only notable finding is the presence of a distinct white forelock at the front of the scalp.

Examination Findings

No specific investigations were conducted for the patient at this time. A clinical diagnosis of Waardenburg syndrome was considered based on the presence of a white forelock in the absence of other physical findings.

Diagnosis

A diagnosis of Waardenburg syndrome was made, due to the presence of white forelock, a characteristic finding of the disease. Although not specific to Waardenburg syndrome, a similar forelock was observed in the daughter who presented with a constellation of symptoms, which are pathognomonic of this condition. Thus, by correlating the findings in these two cases, which comprise this case series, a diagnosis of Waardenburg syndrome was made.

Treatment Plan

Given the patient's age and the lack of significant symptoms, no specific treatment is initiated at this time. However, genetic counseling is recommended for the patient and his family to discuss the implications of Waardenburg syndrome and the disease course and guide managing the condition in their daughter.

Follow-Up

The patient will be scheduled for regular follow-up appointments with an otolaryngologist, audiologist, and ophthalmologist to monitor his hearing, vision, and any potential complications associated with Waardenburg syndrome. Additionally, genetic testing may be considered for the patient and his family to further characterize the underlying genetic mutation responsible for the condition.

Case 2

We report a rare case of a three-month-old female pediatric patient presenting with a constellation of clinical findings of rare and unique genetic syndrome and its sequelae. The patient's guardian sought clinical attention at the pediatric department and outlined the chief complaints. The infant was suffering from constipation for six days. It was also reported that the infant had five distinct episodes of vomiting. The vomitus was described to have a greenish hue, indicating its bilious nature. It was also reported that the infant's abdomen was unusually hard and that she had been refusing feeds. Birth history revealed that in addition to being pre-term, she was also born out of a third-degree consanguineous marriage through a normal vaginal delivery with a birth weight of 2.08 kg. Birth history also brought to light that the patient also had delayed passage of meconium at birth.

**Figure 2:**
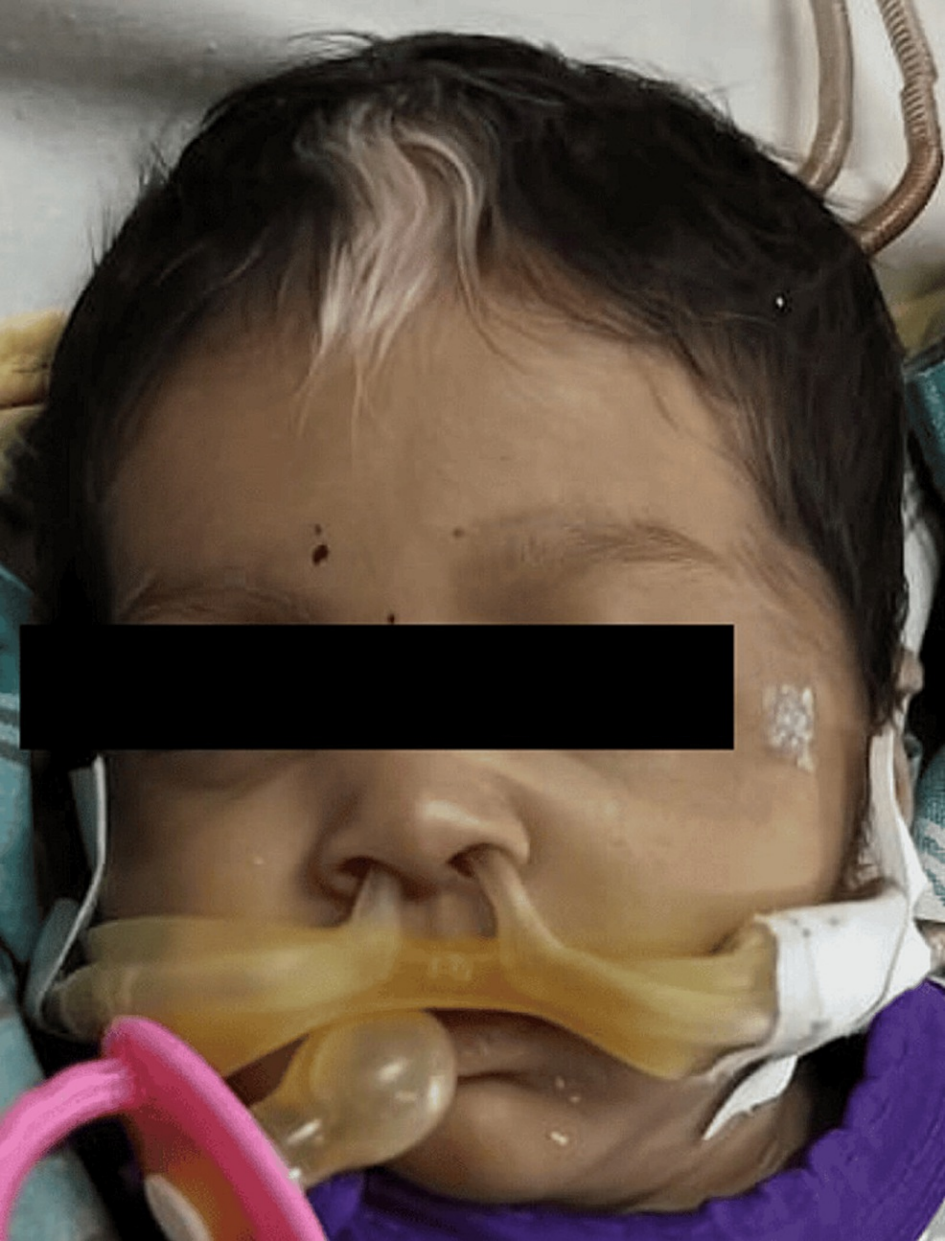
White forelock and broad nasal bridge

**Figure 3:**
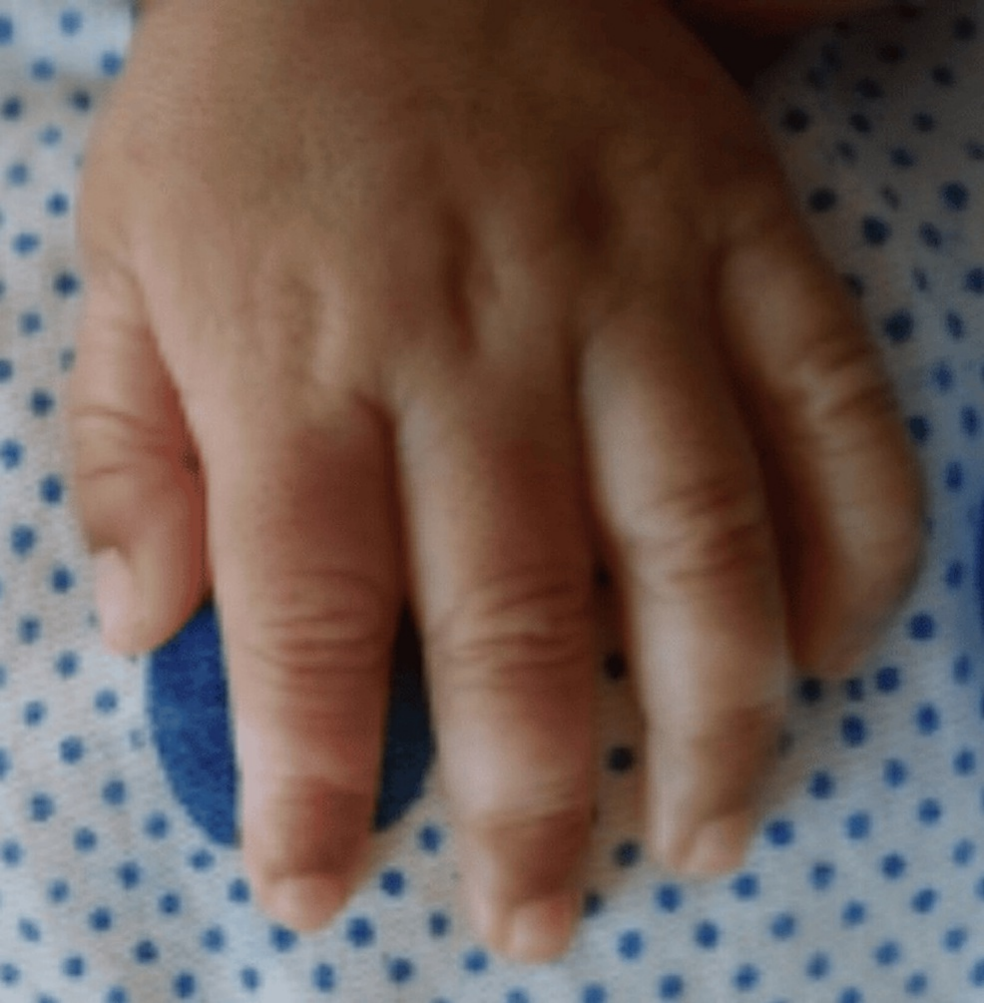
Hypopigmented patches on digits

A general physical examination revealed the presence of a white forelock and a broad nasal septum, as well as hypopigmented patches on the digits (Figures 2, 3). Examination of the gastrointestinal system revealed a grossly distended abdomen. A digital rectal examination was done, which did not reveal any stool or blood on the examining finger. An empty rectum was noted. On deep palpation, there was no organomegaly or any palpable masses. These findings prompted further investigations.

**Figure 4:**
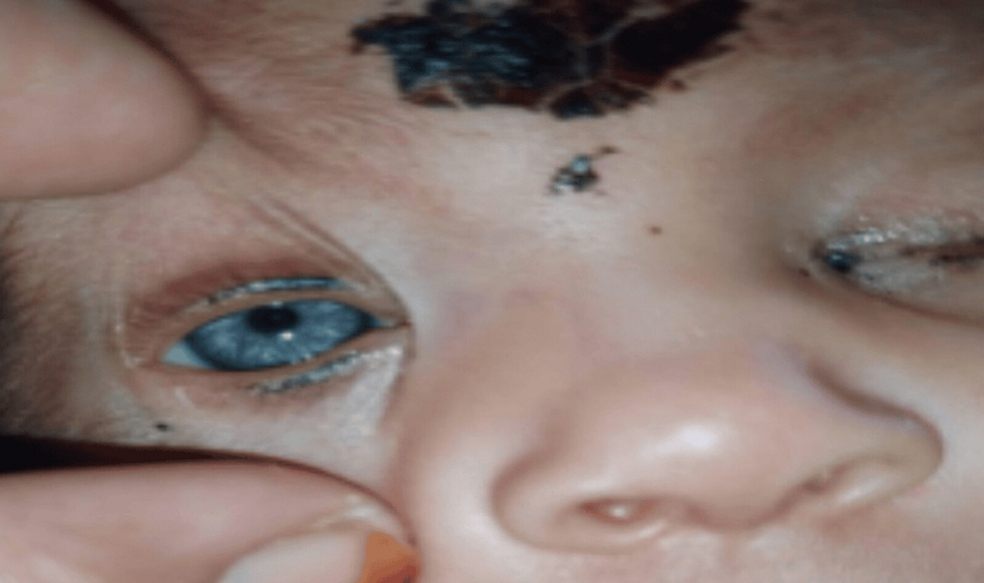
Heterochromia iridis

A plain radiograph of the abdomen was done because of the suspected bowel obstruction, and it revealed the presence of dilated loops of the bowel with air-fluid levels in the left lower quadrant. Abdominal ultrasonography (USG) was unremarkable. Radiographic findings pointed toward a more serious pathology, which prompted a full-thickness rectal biopsy to arrive at a diagnosis. The biopsy demonstrated an absence of ganglion cells, suggestive of Hirschsprung disease. The patient was subsequently referred to the ophthalmology department where the findings of heterochromia iridis were confirmed (Figure 4).

A detailed audiology evaluation revealed an absence of otoacoustic emissions (OAE) and bilateral sensorineural hearing loss. Family history revealed that the patient's father also suffered from a similar genetic condition. However, it was noted that the father did not have the typical finding of heterochromia iridis, which was seen in the neonate.

Diagnosis

Based on the high index of suspicion due to family history, biopsy findings suggestive of Hirschsprung disease, and investigative findings suggestive of bilateral sensorineural hearing loss (SNHL) and heterochromia iridis, a definitive diagnosis of Waardenburg-Shah syndrome was made.

Treatment

The patient was advised surgical management for the Hirschsprung disease and thus underwent a laparoscopic endorectal pull-through procedure. Broad-spectrum antibiotics were administered. The postoperative course was uneventful, and the patient was kept under observation for 10 days until she began appropriate feeds and passed stools. The family was counseled further about the genetic nature of the disease, as well as the disease course and plans for follow-up and a multifaceted care approach.

Within a month, the infant was brought again to the hospital with complaints of cough and cold. A diagnosis of bronchopneumonia was made. The patient was administered appropriate antibiotics and was discharged four days later. The patient was reviewed one month later to monitor her recovery and exhibited adequate weight gain and good recovery.

## Discussion

Waardenburg syndrome is a group of rare congenital genetic syndromes. It was first described by a Dutch ophthalmologist who described a syndrome comprising six distinctive features: lateral displacement of the medial canthus and lacrimal puncta, broad and high nasal root, hypertrichosis of the medial part of the eyebrows, partial or total heterochromia iridis, white forelock, and congenital deaf mutism [12]. It is an autosomal dominant condition, with an incidence of one in 40,000, and accounts for more than 2% of the cases of congenital deafness [2,13]. Also, variations in phenotypic presentation are seen in a few families; this points to the possibility of incomplete gene penetrance of some features in an otherwise autosomal dominant disease [14]. Our case series brings into focus this particular feature of the disease as we highlight the phenotypic feature of heterochromia iridis exhibited by the daughter, but not the father, with the presence of a white forelock in both cases. Thus, the wide array of symptoms with variable severity warrants a patient-centric approach and follow-up care that is tailored to the patient's needs.

The pathogenesis involves abnormal distribution of melanocytes during embryogenesis and mutations in genes that affect the neural crest cells, which accounts for the diverse range of symptoms exhibited by the affected individuals leading to four distinct clinical variants. Types 1 and 2 are the most common. Type 1 WS is characterized by all the aforementioned features and is the prototype of this group of unique syndromes. Type 2 is characterized by pathognomonic findings of sensorineural hearing loss and heterochromia iridum. Type 3 WS has additional musculoskeletal abnormalities. Type 4 WS, which is exhibited in our case series, is the rarest of the four types, presenting with the additional clinical finding of congenital aganglionic megacolon (Hirschsprung disease) with an incidence of 1/1,000,000. Available data suggests that only 50 cases of type 4 WS have been documented, thus highlighting the rarity of this particular subtype [13,15,16].

A high index of clinical suspicion is necessary to consider Waardenburg syndrome as a differential due to its rare incidence. A final diagnosis of Waardenburg syndrome can be difficult, especially without a family history. More importantly, a detailed family history not only aids diagnosis but also warrants a full clinical and audiological examination of the first-degree relatives. This helps to isolate clinical features among other family members, and timely recognition leads to prompt interventions and monitoring, thus reducing the morbidity associated with the disease. This also further underlines the importance of genetic counseling due to the autosomal dominant nature of this disease. Guidance regarding the anticipated symptoms and the necessary therapeutic interventions help to overcome the severity of progressive symptoms of congenital deafness, mutism, and ophthalmologic and musculoskeletal abnormalities and provide timely rehabilitation for the same [17].

Due to the deficits in the available information on this unique group of syndromes, there can be a certain degree of arbitrariness in the diagnostic process, which can be clarified only when the relevant genes are identified and classified by varied mutations to deliver a definitive diagnosis [12]. While research in this regard is ongoing, some pathognomonic mutations have been discovered so far. Types 1 and 3 WS are caused by mutations in the *PAX3* gene, while type 2 WS is caused by mutations in the microphthalmia-associated transcription factor gene. The underlying mutations in type 4 WS include the *EDNRB* and *SOX10* genes. However, family history has exhibited pertinence in this case series as genetic analyses for the mutations specific to each subtype are not attainable in a resource-limited setting [18].

Treatment options for the disease are aimed at correcting and managing the various presentations of the disease. The approach has to be multidisciplinary, given the wide clinical spectrum of the disease. Sensorineural hearing loss must be identified as soon as possible with newborn screening with OAE and BAER [19]. Affected children can be rehabilitated with cochlear implants and associated support. Intestinal obstruction or Hirschsprung disease as seen in our patient can be corrected surgically (endorectal pull-through procedure as done for our patient). The lack of melanocytes and the hypopigmentary lesions present over the body, which are seen in the patients, predispose them to ultraviolet (UV) damage and the potential complication of skin cancer, among other things; therefore, dermatological follow-up periodically with UV protection is imperative [2].

In our case series, we recommended periodic hearings and ophthalmological workup for the father, along with the daughter, to ensure timely measures. We also recommended rehabilitation for the daughter's sensorineural hearing loss. A detailed family history identified that no other first-degree relative suffered from Waardenburg syndrome. Genetic counseling regarding the repetitive occurrence of the disease and its unpredictable course in future offspring was explained to the family members. Thus, genetic counseling, prompt identification of affected members of the family, and future implications of the disease course and its management, which guide the principles of a tailored approach, are what encompasses a holistic approach for this unique syndrome.

## Conclusions

In our case series, we describe two cases of Waardenburg-Shah syndrome from the same family. The diagnosis made based on clinical examination, family history, and investigative findings suggests the presence of type 4 Waardenburg-Shah syndrome, the rarest subtype among this group. Owing to the rarity of the syndrome and limited subtype-specific genetic analysis, it is imperative to broaden research in this area and sharpen the focus on the need for genetic counseling. Also, we stress the importance of cultivating a high index of clinical suspicion for this particular congregation of symptoms, especially until a more cost-effective definitive diagnostic technique is made available for resource-limited settings. A multidisciplinary approach, along with patient-specific treatment modalities and appropriate follow-up care, is warranted in this unique syndromic presentation.
